# A novel adaptive-weight ensemble surrogate model base on distance and mixture error

**DOI:** 10.1371/journal.pone.0293318

**Published:** 2023-10-31

**Authors:** Jun Lu, Yudong Fang, Weijian Han

**Affiliations:** 1 National Center for Applied Mathematics in Chongqing, Chongqing Normal University, Chongqing, China;; 2 School of Mechanical and Vehicle Engineering, Chongqing University, Chongqing, China; 3 Key Laboratory for Lightweight Materials, Nanjing Tech University, Nanjing, China; The Hong Kong Polytechnic University, HONG KONG

## Abstract

Surrogate models are commonly used as a substitute for the computation-intensive simulations in design optimization. However, building a high-accuracy surrogate model with limited samples remains a challenging task. In this paper, a novel adaptive-weight ensemble surrogate modeling method is proposed to address this challenge. Instead of using a single error metric, the proposed method takes into account the position of the prediction sample, the mixture error metric and the learning characteristics of the component surrogate models. The effectiveness of proposed ensemble models are tested on five highly nonlinear benchmark functions and a finite element model for the analysis of the frequency response of an automotive exhaust pipe. Comparative results demonstrate the effectiveness and promising potential of proposed method in achieving higher accuracy.

## Introduction

Computational simulation models have been widely used to as a substitute for costly and time-consuming physical experiments [1]. In spite of sustained growths in computer capability and speed, the enormous computational cost still makes it impractical to rely solely on highly expensive simulation models for design and optimization [2]. Surrogate modeling, as a cost-effective alternative to expensive simulation models, is a special case of supervised machine learning applied in the field of engineering design [3–6]. Over the past few decades, there is a growing interest in developing surrogate modeling techniques in many different engineering disciplines [7–10]. There are a lot of commonly used metamodels, include Polynomial Regression models [11], Support Vector Regression models [12, 13], Radial Basis Function interpolation models [14, 15], Kriging/Gaussian Process models [16–18], Artificial Neural Network models [19, 20], *et al*.

A more detailed overview on various surrogate modeling techniques can be found in Refs. [21, 22]. Han [23] *et al.* conducted comparative analysis of different surrogate modeling methods. Prior studies have shown that ANN is better suited for ultra-high-dimensional problems with over 1000 variables. RBF is suitable for approximating high-order nonlinear functions, while KRG is suitable for fitting functions with high dimensionality and low nonlinearity. PRS exhibits good approximation capabilities for low-dimensional, low nonlinearity functions, or functions with data noise. When training samples are limited, SVR demonstrates better fitting performance and is more suitable for handling low-dimensional problems. Normally, there is no single surrogate model that can provide the best prediction accuracy for every scenario.

To ease this problem, the ensemble surrogate model, constructed by combining multiple individual surrogate models, have been widespread concerned [24, 25]. The ensemble surrogate model can leverage the advantages of each individual model at a lower computational cost and extract trend information from the design space. In other words, it would be beneficial to combine multiple surrogate models to enhance the accuracy of the response predictions [26]. There is a growing interest in developing ensemble surrogate models. For instance, based on the principles of neural network modeling, Zerpa *et al.* [27] proposed an ensemble surrogate model in which the weights of the component models are calculated based on cross-validation mean squared errors. The smaller the cross-validation error of a component model, the larger its corresponding weight. Goel *et al.* [28] proposed a weighted ensemble surrogate model based on predicted residual error sum of squares. Viana *et al.* [24] and Acar and Rais-Rohani [29] introduced an optimization strategy to optimize the weights of the component surrogate models. Based on Dempster-Shafer theory, Muller *et al.* [30] developed a weight calculation method to construct an ensemble surrogate model. In their work, an evidence combination rule is adopted to comprehensively consider various error metrics of the component surrogate models.

It is important to point out that the previously mentioned ensemble surrogate model is fix-weight ensemble surrogate model(FWESM) across the entire design space. The main shortcoming of FWESM is that they fail to adaptively adjust the weight of component models based on the local error of those component models. In recent years, the studies on adaptive weight ensemble surrogate model(AWESM), which is more related to the method to be developed in this work, have attracted significant attention to many researchers. The objective of employing adaptive weights is to dynamically allocate greater importance or influence to the component models that perform better, while diminishing the effect of weaker models. In this respect, Sanchez *et al.* [31] proposed an AWESM in which the weight factors of the component surrogate models are calculated based on the prediction variance of the nearby training samples. Zhang *et al.* [32] proposed a unified ensemble of surrogates (UES) method, which uses a linear combination of a global measure-based weight calculation and a local measure-based weight calculation to define weight factors. Huang *et al.* [33] proposed a robust ensemble surrogate model based on a hybrid error measurement. Indeed, when building an AWESM, it is crucial to account for the spatial relationship between the sample to be predicted and the entire training dataset. Furthermore, one must consider the fact that interpolation models exhibit better prediction accuracy in proximity to training sample points. Motivated from this finding, the main objective of the paper is to develop a new AWESM capable of providing a higher accuracy of the response predictions with limited samples.

This paper presents a novel AWESM that considers several critical factors in its weight calculation. These factors include the spatial relationship between prediction samples and training samples, the assessment of mixture errors in the component surrogate model, and characteristics that resemble interpolation models. Our approach stands out from other methods in the literature in the following ways: Firstly, we calculate the initial weights for component surrogate models based on a combination of global and local errors. These initial weights are then refined according to the distance between the prediction sample and the training samples. Furthermore, we introduce a weight adjustment factor to fine-tune the weights of the fitting models, thereby imbuing our ensemble model with interpolation model characteristics. Secondly, the weight adjustment factor is determined by considering the distance between a prediction sample and its two nearest training samples. Ultimately, the final weights of the component surrogate models are computed by combining the initial weights and the weight adjustment factor.

The remainder of this paper is organized as follows: Firstly, the commonly used surrogate models, encompassing individual surrogate models and ensemble surrogate models, are introduced. Followed by the illustration of the proposed novel adaptive ensemble surrogate model. Then, engineering and numerical studies are carried out to evaluate the performance of the proposed method. The conclusions are provided in the last section.

## Ensemble surrogate models

The surrogate model is a type of supervised learning algorithm that is used to approximate the input-output relationship of a computationally expensive numerical model. The surrogate model can replace time-consuming simulation calculations during the optimization process, allowing for the completion of computationally intensive global optimization within a short period. Commonly used individual surrogate models in engineering structure design includes Polynomial Regression model (PR), Support Vector Regression model (SVR), Kriging/Gaussian Process model (KRG/GP) and Radical Basis Function interpolation model (RBF). The characteristic of these models are summarized in Table 1. Different surrogate models can capture different features and patterns in the data, making them suitable for various engineering problems. Ensemble surrogate model can leverage the advantages of multiple individual surrogate models to enhance the overall forecasting performance, mitigate the risk of over-fitting associated with a single model, and deliver more consistent forecasting results.

**Table 1:** A summary of the commonly used individual surrogate models.

Models	Characteristics
Polynomial regression	Easy construction and implementation Suitable for applications with few parameter variables Suitable for applications with random noise Explicit expression for input-output relationship
Kriging/Gaussian Process	Suitable for nonlinear problems Beneficial for prediction variance Beneficial for complex correlation functions Time-consuming model training
Support Vector Regression	Simple structure, strong generalizability Able to solve small sample regression problems Suitable for nonlinear problems
Radical Basis Function	Suitable for highly nonlinear problems Suitable for high-dimensional input problems Suitable for modeling with limited training sample data

An ensemble surrogate model, also known as a mixture surrogate model, hybrid surrogate model or integrated surrogate model, is generally composed of several trained individual surrogate models combined through weighted aggregation. It has the following form:

$$\begin{array}{c} f_{e}\left(x\right)=\sum_{i=1}^{m}w_{i}\hat{f_{i}}\left(x\right),\;\sum_{i=1}^{m}w_{i}=1 \end{array}$$ (1)

In the equation, *f*_*e*_(***x***) represents the ensemble surrogate model, *m* is the number of component surrogate models used to construct the ensemble surrogate model, $\hat{f_{i}}\left(x\right)$ is the *i*^*th*^ component surrogate model, and *w*_*i*_ is a non-negative weight coefficient function associated with the corresponding component surrogate model. Depending on whether the weight coefficients vary with the location change of prediction sample in the design space or not, the ensemble surrogate model can be classified as either a FWESM or an AWESM.

### Fixed-weight ensemble surrogate model

The weights *w*_*i*_ of each component surrogate model in the FWESM remain constant throughout the entire design space. Existing research has focused on determining the weights of the component surrogate models based on their global accuracy. The component surrogate models with higher global accuracy are assigned larger weights, while those with lower accuracy are assigned smaller weights or even zero weights.

In 2007, Goel *et al.* proposed the PRESS weighted surrogate (PWS) model, which calculates the weights of component surrogate models based on the predicted residual error sum of squares (PRESS) [28]. The weights of the component surrogate models in the PWS model, denoted as $w_{i}^{pws}$, are calculated as follows:

$$\begin{array}{c} \begin{array}{c} w_{i}^{pws}=w_{i}^{*}/\sum_{i=1}^{m}w_{i}^{*}\\ w_{i}^{*}={\left(E_{i}+\eta \bar{E}\right)}^{\beta },\bar{E}=E_{i}/\sum_{i=1}^{m}E_{i}\\ \eta \leq 1,\beta \leq 0 \end{array} \end{array}$$ (2)

where *E*_*i*_ represents generalized mean squared error of the *i*^*th*^ component surrogate model, $\bar{E}$ is the mean value of *E*_1_, *E*_2_, ⋯, *E*_*m*_. *α* and *β* are control parameters that regulate the influence of the mean of individual component surrogate models and the mean of all component surrogate models on the ensemble surrogate model. Research has shown that setting *η* = 0.05 and *β* = −1 provides good stability for the PWS model. The generalized mean square error (GMSE) is obtained through leave-one-out cross-validation (LOOCV) and is defined as follows:

$$\begin{array}{c} GMSE=\frac{1}{n}\sum_{i=1}^{n}{\left({\hat{y}}^{-i}\left(x_{i}\right)-y_{i}\left(x_{i}\right)\right)}^{2} \end{array}$$ (3)

where ${\hat{y}}^{-i}\left(x_{i}\right)$ represents the prediction of the surrogate model for the sample ***x***_*i*_, where the *i*^*th*^ training sample is excluded from the model training.

In 2009, Acar and Rais-Rohani proposed a method called BestPRESS to calculate the weights of the component surrogate models. This method optimizes the weight coefficients to minimize the PRESS of the ensemble surrogate model [29]. The weights $w_{i}^{bpr}$ of the component surrogate models are obtained by solving the following optimization problem:

$$\begin{array}{c} \begin{array}{cc} \text{Find:}\; & w_{i}^{bpr}\\ \text{Minimize:}\; & \sqrt{\frac{1}{n}\sum_{k=1}^{n}\left(y\left(x_{k}\right)-\sum_{i=1}^{m}w_{i}{\hat{y}}_{i}^{-k}{\left(x_{k}\right)}^{2}\right)}\\ \text{Subject}\;\text{to:}\; & \sum_{i=1}^{m}w_{i}=1 \end{array} \end{array}$$ (4)

In 2009, Viana *et al.* proposed an optimization-based method called Optimal Weighted Surrogate (OWS) model to calculate the weights of the component surrogate models [24]. This method minimizes the cross-validation error by considering the covariance matrix formed by the prediction residuals of the component surrogate models. The weights $w_{i}^{ows}$ of the component surrogate models are obtained by solving the following optimization problem:

$$\begin{array}{c} \begin{array}{cc} \text{Find:}\; & w_{i}^{ows}\\ \text{Minimize:}\; & MSE=w^{T}Cw\\ \text{Subject}\;\text{to:}\; & \sum_{i=1}^{m}w_{i}=1 \end{array} \end{array}$$ (5)

where ***C*** is the covariance matrix. $C_{i,j}=\frac{1}{N}{cv}_{i}^{T}{cv}_{j}$ represents the covariance between *i*^*th*^ and *j*^*th*^ component surrogate models derived from cross-validation errors.

### Adaptive-weight ensemble surrogate model

The AWESM, also known as variable weighted ensemble surrogate, refers to a type of ensemble surrogate model where the contribution of component surrogate models to the ensemble model varies at different locations in the design space. The calculation rules for the weights in the AWESM typically take into account the local prediction accuracy of the component surrogate models.

In 2008, Sanchez *et al.* [31] proposed a weight calculation strategy for the AWESM based on the Prediction Variance (PV), which serves as a local error indicator. In their proposed method, the weight of a component surrogate model at a prediction sample ***x*** is given by:

$$\begin{array}{c} w_{i}^{pv}=\frac{1/PV_{i}\left(x\right)}{\sum_{i=1}^{m}\frac{1}{PV_{j}\left(x\right)}} \end{array}$$ (6)

where *PV*_*i*_(***x***) represents the prediction variance of the *i*^*th*^ component surrogate model at the prediction sample ***x***. It is computed as:

$$\begin{array}{c} PV_{i}\left(x\right)=\frac{1}{k-1}\sum_{l=1}^{k}{\left(y\left(x_{l}\right)-{\hat{y}}_{i}^{-l}\left(x_{l}\right)\right)}^{2} \end{array}$$ (7)

where the term *k* denotes the number of neighboring points of ***x***, and based on testing, Sanchez *et al.* set its value to 3. The term ${\hat{y}}_{i}^{-l}\left(x_{l}\right)$ represents the leave-one-out prediction of the *i*^*th*^ component surrogate model at the neighboring point ***x***_*l*_.

In 2021, Huang *et al.* [33] proposed the ensemble of surrogate based on hybrid error metric (ESHEM) for weight calculation. The weights can be computed using the following equation:

$$\begin{array}{c} w_{i}^{eshem}=\frac{1/E_{i}\left(x\right)}{\sum_{j=1}^{m}\frac{1}{E_{j}\left(x\right)}},\;E_{i}\left(x\right)=GMSE_{i}\times PV_{i}\left(x\right) \end{array}$$ (8)

where *GMSE*_*i*_ represents the generalized mean square error calculated through leave-one-out validation for the *i*^*th*^ component surrogate model. When computing the prediction variance of neighboring points *PV*_*i*_(***x***) using Eq 6, only the neighboring points with Euclidean distance less than the average Euclidean distance between all training samples and the prediction sample are considered.

## Materials and methods

More recently, Zhou *et al.* [34] pointed out that the weight combination strategy based on global error ensures the accuracy of the ensemble surrogate model across the entire design space, while the weight combination strategy based on local error serves as a diversity metric for individual surrogate models at different positions in the design space. In the design space, neighboring points often have similar values. Therefore, when the prediction sample is far from the main region where most sample points are located, its prediction value relies more on the values of adjacent sample points. In other words, in this case, local error measurement plays a dominant role in determining the weight factors. Conversely, global error measurement accounts for a larger proportion when the prediction sample is exactly located in the main region.

### Illustration of the proposed method

In this paper, a distance and mixture error based ensemble surrogate model (DMEES) is proposed. The proposed DMEES incorporates the position of the prediction sample, the mixture error metric, and the leaning features of the component surrogate models. Considering the global error, local error, and the spatial relationship between prediction sample and training samples, the initial weights can be calculated using the following equation:

$$\begin{array}{c} \begin{array}{c} w_{i}^{inital}=w_{i}^{global}*\left(1-\lambda \right)+w_{i}^{pv}*\lambda \end{array} \end{array}$$ (9)

where $w_{i}^{initial}$ represents the initial weight of the *i*^*th*^ component surrogate model at the prediction sample ***x***. $w_{i}^{global}$ is the weight combination factor that considers global error, which can be calculated using Eq 4. $w_{i}^{pv}$ is the weight factor that considers local error, which can be calculated using Eq 6. When calculating the prediction variance *PV*_*i*_(***x***) in Eq 6, only the neighboring points whose Euclidean distance to the prediction sample is smaller than the average Euclidean distance among all training samples are considered.

The parameter λ ∈ [0, 1] is a distance-related parameter. It controls the influence of global error measurement and local error measurement based on the distance relationship between prediction sample and trainning sanples. λ can be calculated using the following equation:

$$\begin{array}{c} \begin{array}{c} \lambda =\int_{-\infty }^{r}\frac{1}{\sqrt{2\pi }\left(r_{1}/3-r_{2}/3\right)}e^{\frac{r-r_{1}}{2{\left(r_{1}/3-r_{2}/3\right)}^{2}}}dr \end{array} \end{array}$$ (10)

*r* represents the average Euclidean distance between the prediction sample ***x*** and the training samples, which reflects the distance between the sample to be predicted and the main area where most samples are located. *r*_1_ is the average Euclidean distance of training samples, indicating the uniformity of the distribution of training samples. *r*_2_ is the minimum average Euclidean distance of a training sample to other training samples, indicating the density of the training samples. For a given training set ***S*** = (***x***_1_, *y*_1_), (***x***_2_, *y*_2_), (***x***_3_, *y*_3_), …, (***x***_*n*_, *y*_*n*_), where $x_{i}\in R^{n_{d}}$, *r*, *r*_1_ and *r*_2_ are expressed as following equation.

$$\begin{array}{c} \begin{array}{cc} r & =\frac{1}{n}\sum_{i=1}^{n}dis(x,x_{i})\\ r_{1} & =\frac{2}{n(n-1)}\sum_{i=1}^{n-1}\sum_{j=i+1}^{n}dis(x_{i},x_{j}),\\ r_{2} & =min(\frac{1}{n-1}\sum_{j=1}^{n}dis\left(x_{i},x_{j}\right)),\text{where}\;i\neq j \end{array} \end{array}$$ (11)

**Fig 1:**
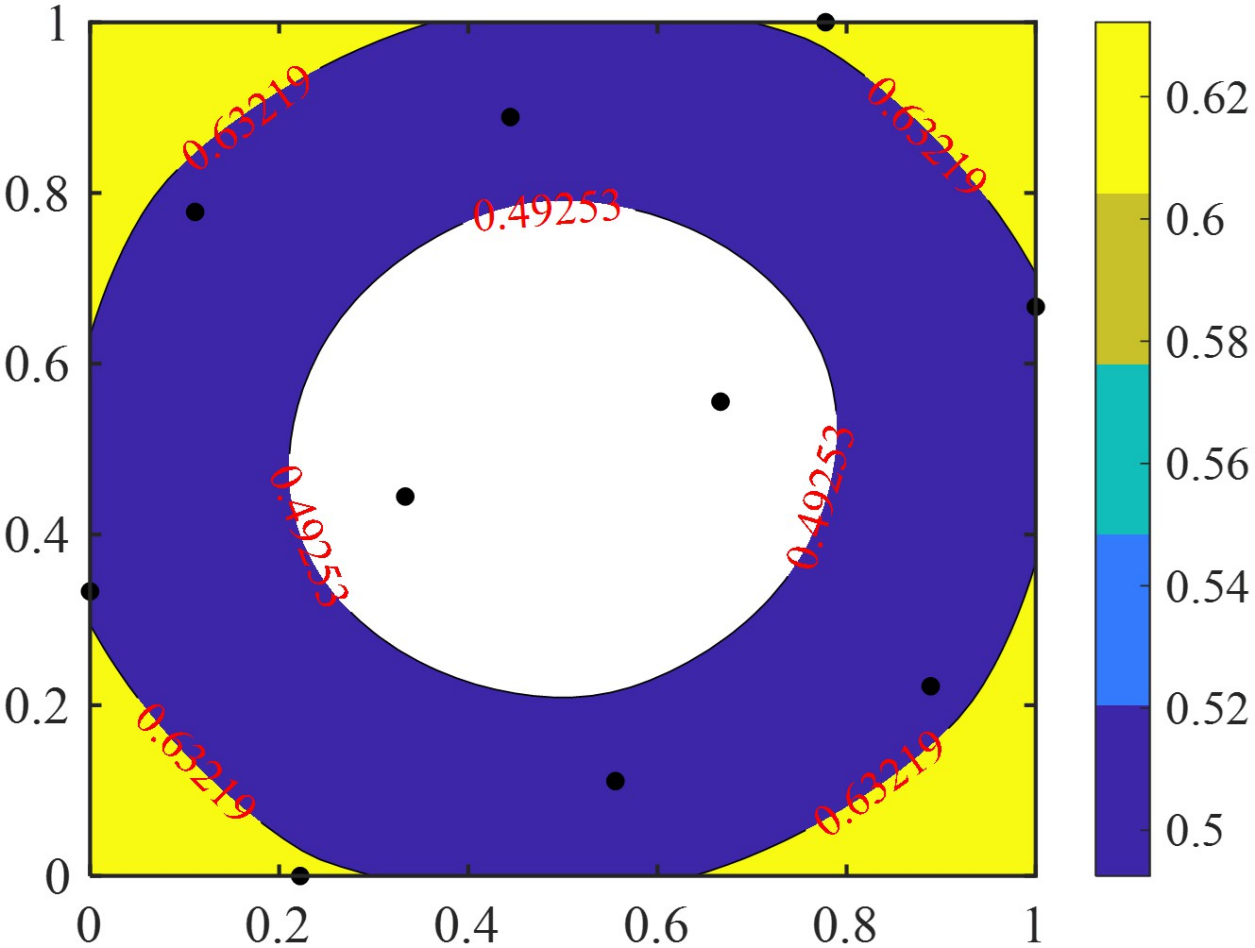
Contour plot of the average Euclidean distance between the prediction sample *x* and a given training set.

**Fig 2:**
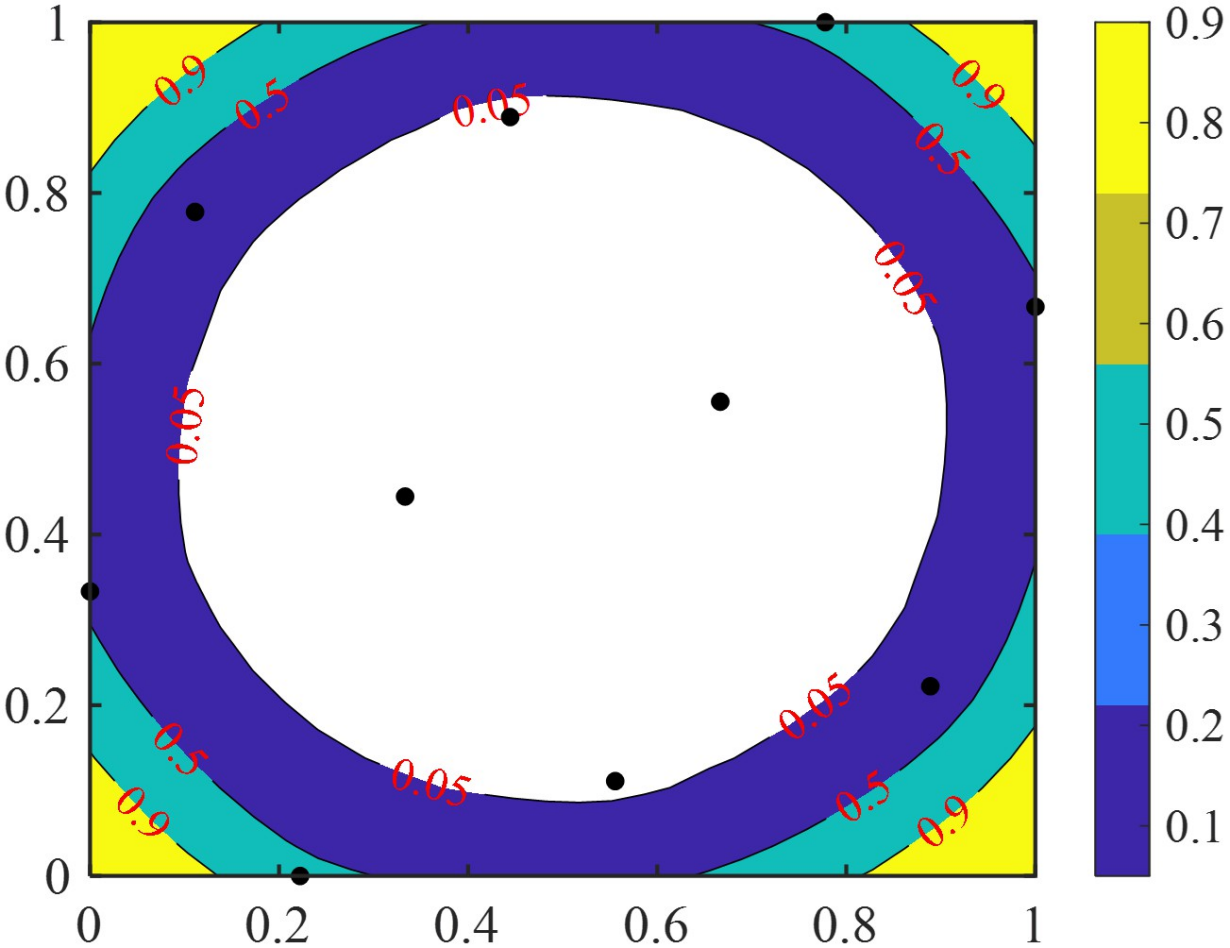
Contour plot of parameter λ with a given training set.

Fig 1 illustrates the average Euclidean distance (*r*) between the prediction sample, ***x*** and a given set of training samples. The black dots in the figure are training samples, and the contour values from large to small are *r*_1_ and *r*_2_, respectively. It can be seen from the figure that the samples to be predicted in the edge region have larger *r* values. Fig 2 shows the distribution of the parameter λ for the given set of training samples. It can be observed that as the prediction sample gets closer to the edge region, the value of λ increases, indicating that the weight calculation of the ensemble surrogate model relies more on the errors of neighboring points.

The current weight calculation methods for ensemble surrogate models have not effectively taken into account the learning characteristics of component surrogate models. The commonly used surrogate models can be classified into two types: fitting models and interpolation models. Both types establish approximation functions based on a finite set of training samples to represent the relationships between the input and output variables. Fitting models typically minimize the total error at the training samples through regression equations, while interpolation models construct approximation functions based on spatial distances to ensure that the functions pass through all training samples.

**Fig 3:**
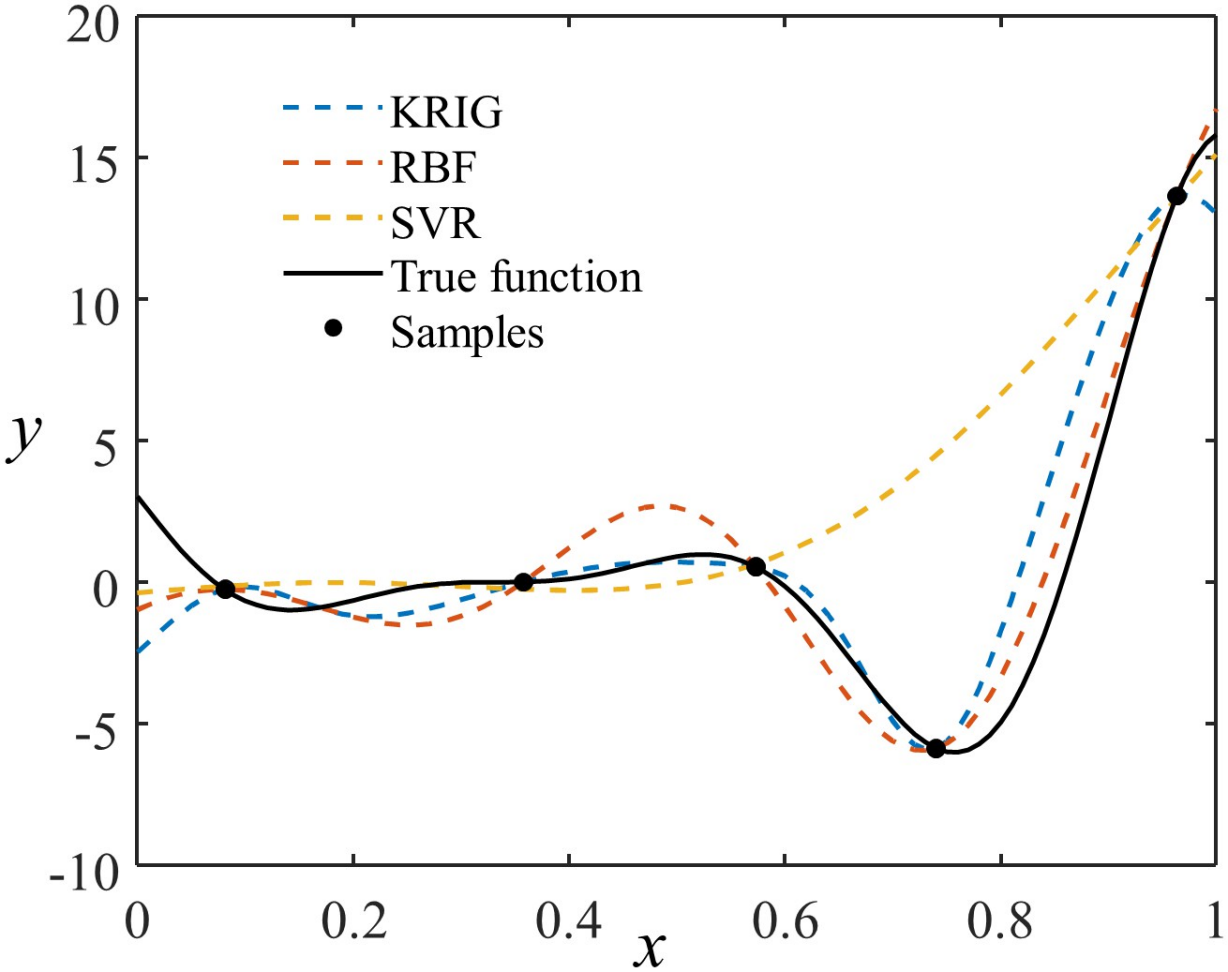
The approximation of Forrester functions by interpolation and fitting models. The KRG model uses first-order polynomials and Gaussian correlation functions, the RBF model uses Gaussian kernel functions, and the SVR model uses Gaussian kernel functions.

Fig 3 shows the approximations of the Forrester function [35] using SVR, KRG, and RBF based on five given sample points. SVR and PR models are typical fitting models, while KRG and RBF models are typical interpolation models. When using interpolation models, the prediction of a given prediction sample is more influenced by the neighboring points if it is closer to a training sample. Therefore, as interpolation models, KRG and RBF models exhibit higher accuracy near the existing sample points. In this study, we take this factor into consideration when constructing the ensemble surrogate model. We adjust the weights of the fitting models by introducing a fitting model weight adjustment factor, enabling the ensemble surrogate model to possess interpolation model characteristics. The fitting model weight adjustment factor *α* can be calculated using the following equation,

$$\begin{array}{c} \begin{array}{c} \alpha =\text{sin}\;\left(\frac{d_{1}}{d_{2}}*\frac{\pi }{2}\right) \end{array} \end{array}$$ (12)

where *d*_1_ and *d*_2_ represent the Euclidean distances from the sample to be predicted, ***x*** to the nearest and second-nearest training samples, respectively. As ***x*** gets closer to a specific training sample, the fitting model weight adjustment factor *α* approaches zero. After incorporating the fitting model weight adjustment factor, the final weight $w_{i}^{final}$ for the DMEES model is given by,

$$\begin{array}{c} \begin{array}{c} w_{i}^{final}=\frac{w_{i}^{adjust}}{\sum_{j=1}^{m}w_{i}^{adjust}},\\ w_{i}^{adjust}=\{\begin{array}{ccc} & w_{i}^{inital}*\alpha , & \text{If}\;\text{model}\;\text{is}\;\text{a}\;\text{fitting}\;\text{model}\;\\ & w_{i}^{inital}, & \text{Otherwise}\;\;\;\;\;\;\;\;\;\;\;\;\;\;\;\;\;\;\;\;\;\; \end{array} \end{array} \end{array}$$ (13)

Based on the final weighting factors of the component surrogate models, the proposed DMEES model predicts the value at the prediction sample ***x*** as follows:

$$\begin{array}{c} f_{e}\left(x\right)=\sum_{i=1}^{m}w_{i}^{final}\hat{f_{i}}\left(x\right). \end{array}$$ (14)

According to Eq 12, when *d*_1_ = 0, it means the prediction sample coincides with a training sample point, the adjustment factor *α* for the fitting model weights becomes 0. Consequently, the component surrogate model with fitting model characteristics has a weight of 0 at that prediction sample. Therefore, the DMEES model also possesses the feature of an interpolation model, a zero prediction error at the training sample points.

### Flowchart of the proposed method

**Fig 4:**
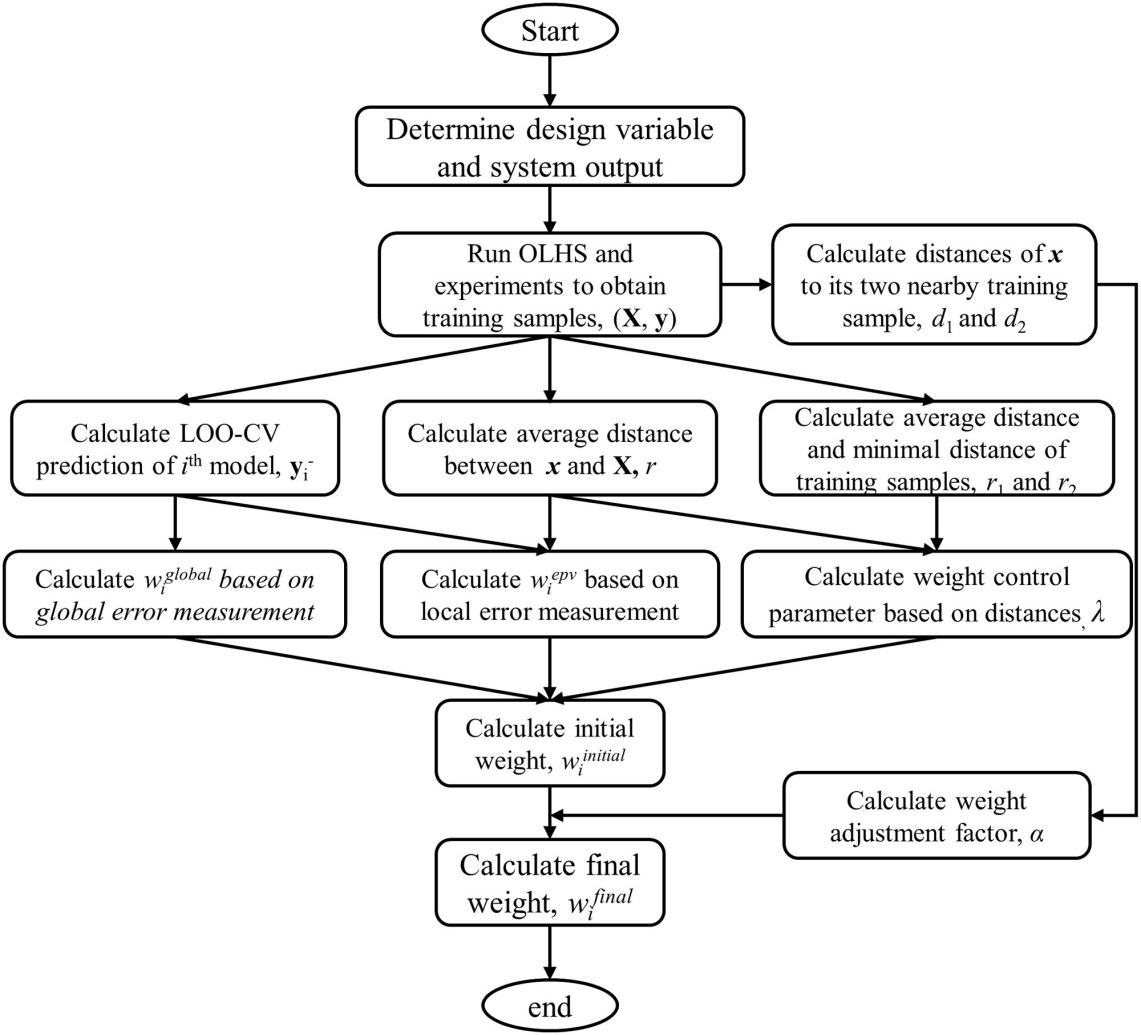
Process for calculating the weight factor of the DMEES model at a prediction sample.

Fig 4 illustrates the process of calculating the weights of the component surrogate models at a given prediction sample ***x*** when using DMEES. After determining the ranges of design variables and the system output, the training sample input matrix ***X*** can be obtained using the Optimal Latin Hypercube Sampling (OLHS) algorithm. The simulation experiments are then conducted to obtain the training sample output vector ***y***. Based on the available training samples, the weights of each component surrogate model at the prediction sample can be calculated using the following steps:

Step 1: Using the leave-one-out cross-validation (LOO-CV) approach, obtain the outputs $y_{i}^{-}$ corresponding to the *i*^*th*^ component surrogate models (KRG, RBF, SVR).

Step 2: With the support of the data ***y*** and $y_{i}^{-}$, calculate the weights of the component surrogate models based on the global error measurement using Eq 4, that is, $w_{i}^{global}=w_{i}^{bpr}$.

Step 3: Calculate the average Euclidean distance *r*_1_ and the minimum average Euclidean distance of a training sample to other training samples *r*_2_ with the known ***X***. Calculate the average Euclidean distance *r* between the prediction sample ***x*** and the training samples ***X***. Given the distance data *r*_1_, *r*_2_, and *r*, calculate the distance control parameter λ for the weights of the component surrogate models using Eq 10.

Step 4: With the known ***y***, $y_{i}^{-}$, and *r*, calculate the weights of the component surrogate model based on the local error using Eq 6, namely, $w_{i}^{pv}$. When evaluating Eq 6, the value of *k* represents the number of training samples whose Euclidean distance from the prediction sample is smaller than the average Euclidean distance *r*_1_ between all training samples.

Step 5: Given the weights of the component surrogate models based on global error $w_{i}^{global}$, the weights of the component surrogate models based on local error $w_{i}^{pv}$, and the weight control parameter based on distance λ, calculate the initial weights of the component surrogate model using Eq 9.

Step 6: Calculate the Euclidean distances *d*_1_ and *d*_2_ between the prediction sample ***x*** and the nearest and second nearest samples in the training samples ***X***. Compute the weight adjustment factor for the fitting models *α* based on Eq 12, and determine the final weights of the component surrogate models $w_{i}^{final}$ based on Eq 13.

## Performance test of various surrogate models

This section will test the performance of the proposed method through several numerical and engineering examples. The proposed method will be compared with some surrogate models mentioned in this paper. The component surrogate models for constructing the ensemble surrogate model include the KRG model with a first-order polynomial and Gaussian correlation function, the RBF model with a Gaussian kernel function, and the SVR model with a Gaussian kernel function. The RBF and KRG models are implemented using the Modularized Surrogate Model Toolbox, a surrogate modeling MATLAB toolkit developed by Muller *et al.* [36]. The hyperparameters of the surrogate model use the default parameter settings, and an internal tuning algorithm is integrated into the toolbox, ensuring that the approximate model achieves high accuracy in most cases. The SVR model is implemented using the Libsvm toolkit developed by Chih-Jen Lin *et al.* [37]. Since the regularization parameter and kernel bandwidth parameter of the SVR model have a significant impact on the model’s generalization performance, a grid search method is used to optimize these parameters during the actual modeling process.

Since relying solely on a single error metric cannot objectively reflect the performance of the model, two commonly used model error metrics, namely the Maximum Absolute Error (MAE) and Root Mean Square Error (RMSE), are used to evaluate the accuracy of the models. MAE and RMSE are formulated as follows:

$$\begin{array}{c} \begin{array}{cc} \text{RMSE}= & \sqrt{\frac{1}{N}\sum_{i=1}^{N}{\left(\hat{y_{i}}-y_{i}\right)}^{2}}\\ \text{MAE}= & \frac{1}{N}\sum_{i=1}^{N}abs\left(\hat{y_{i}}-y_{i}\right) \end{array} \end{array}$$ (15)

where *N* represents the number of test samples, *y*_*i*_ represents the actual output value of the *i*^*th*^ sample, ${\hat{y}}_{i}$ represents the output value predicted by the surrogate model, and $\bar{y}$ represents the mean value of the actual output values for all test samples. RMSE and MAE are served as the global error measurement and local error measurement of a surrogate model, respectively [38, 39]. The smaller the values of RMSE and MAE, the higher the accuracy of the surrogate model.

### Nonlinear function test

Five nonlinear functions are selected to test the approximation capability of the proposed approach. The selected test functions are the Six-hump camel-back function, Ackley function, Hartman function, Extended Rosenbrock function, and Dixon-Price function [40, 41]. The five test functions are numbered from 1 to 5 in order. Table 2 provides the expressions of the test functions and information about the variable ranges. The parameters related to the Hartman function can be found in [40].

**Table 2:** Information of test functions.

No.	Dimensions	Expression	VVariable range
1	2	$y=\left(4-2.1x_{1}^{2}+\frac{x_{1}^{4}}{3}\right)x_{1}^{2}+x_{1}x_{2}+\left(4x_{2}^{2}-4\right)x_{2}^{2}$	*x*_1_ ∈ [−3, 3] *x*_2_ ∈ [−2, 2]
2	4	$\begin{array}{cc} y= & 20\;\text{exp}\;(-0.2\sqrt{\frac{1}{4}\sum_{i=1}^{4}x_{i}^{2}})+\text{exp}\;\left(1\right)\\ & -\text{exp}\;(\frac{1}{4}\sum_{i=1}^{4}\text{cos}\;\left(2\pi x_{i}\right))+20 \end{array}$	*x*_*i*_ ∈ [−2, 2]
3	6	$y=-\sum_{i=1}^{4}c_{i}\;\text{exp}\;(-\sum_{j=1}^{6}a_{ij}{\left(x_{j}-p_{ij}\right)}^{2})$	*x*_*i*_ ∈ [0, 1]
4	9	$y=-\sum_{i=1}^{8}\left[100{\left(x_{i+1}-x_{i}^{2}\right)}^{2}+\left(1-x_{i}^{2}\right)\right]$	*x*_*i*_ ∈ [−5, 10]
5	12	$y={\left(x_{1}-1\right)}^{2}-\sum_{i=2}^{12}\cdot {\left(2x_{i}^{2}-x_{i-1}\right)}^{2}$	*x*_*i*_ ∈ [−10, 10]

Taking into account the influence of sample size on the approximation ability of the surrogate model, we set three different sizes of training samples. Sample sizes for small, medium, and large cases are set as 6*n*_*d*_, 12*n*_*d*_, and 24*n*_*d*_, respectively, in which *n*_*d*_ represents the number of independent variables. The test samples are generated using the OLHS method with a sample size of 500. Due to the significant magnitude differences in the outputs of different test functions, in order to facilitate the comparison and analysis of model performance, the test sample outputs and surrogate model outputs are normalized based on the maximum and minimum values of the test sample outputs. Then, the model errors could be calculated based on the normalized data. The robustness of the surrogate model, when trained with different training sample sets, is tested by generating 100 different sets of training samples using the Matlab function ‘lhsdesign’ with different random seed settings. Table 3 presents the median values for the error measurements of the surrogate models when trained with different training sample sets. The gray background in the table represents individual surrogate models, while the white background represents ensemble surrogate models. The best-performing individual surrogate model and the best-performing ensemble surrogate model are highlighted in bold in the table.

**Table 3:** Median values of approximation error for surrogate models under different training samples.

Sample size	Error measurements	KRG	RBF	SVR	PWS	OWS	BestP	PV	ESHEM	DMEES
Six-hump camel-back function										
**Small**	**RMSE**	0.438	**0.419**	0.422	0.410	0.419	0.419	0.410	0.410	**0.391**
	**MAE**	0.832	0.768	**0.744**	0.745	0.748	0.746	0.744	0.739	**0.689**
**Medium**	**RMSE**	0.372	**0.354**	0.355	0.344	0.349	0.350	0.343	0.342	**0.337**
	**MAE**	0.686	0.656	**0.573**	0.595	**0.562**	0.582	0.607	0.594	0.596
**Large**	**RMSE**	**0.184**	0.290	0.200	0.209	0.194	**0.192**	0.207	0.198	0.208
	**MAE**	**0.244**	0.503	0.266	0.293	0.256	**0.250**	0.296	0.265	0.292
Ackley function										
**Small**	**RMSE**	0.422	0.382	**0.371**	0.375	0.371	0.372	0.375	0.373	**0.367**
	**MAE**	0.581	0.485	**0.468**	0.484	0.467	0.467	0.487	0.474	**0.457**
**Medium**	**RMSE**	0.380	0.396	**0.352**	0.363	0.354	**0.352**	0.363	0.359	0.356
	**MAE**	0.502	0.548	**0.435**	0.451	0.439	0.439	0.454	0.449	**0.427**
**Large**	**RMSE**	0.374	0.415	**0.335**	0.352	0.335	**0.335**	0.354	0.346	0.346
	**MAE**	0.484	0.609	**0.411**	0.445	0.413	**0.413**	0.445	0.437	0.422
Hartman function										
**Small**	**RMSE**	0.372	**0.364**	0.365	0.360	0.364	0.362	0.359	0.360	**0.337**
	**MAE**	0.830	**0.759**	0.805	0.789	0.792	0.788	0.785	0.789	**0.623**
**Medium**	**RMSE**	0.338	**0.336**	0.343	0.332	0.335	0.333	0.331	0.331	**0.308**
	**MAE**	0.743	**0.683**	0.740	0.719	0.717	0.726	0.713	0.720	**0.578**
**Large**	**RMSE**	**0.292**	0.299	0.314	0.292	0.297	0.294	0.291	0.293	**0.274**
	**MAE**	**0.594**	0.571	0.654	0.597	0.581	0.593	0.595	0.596	**0.501**
Extended Rosenbrock function										
**Small**	**RMSE**	0.351	**0.311**	0.452	0.353	0.312	0.311	0.349	0.340	**0.247**
	**MAE**	0.489	**0.415**	0.773	0.532	0.430	0.424	0.534	0.509	**0.176**
**Medium**	**RMSE**	0.304	**0.285**	0.435	0.324	0.287	0.287	0.322	0.310	**0.229**
	**MAE**	0.417	**0.365**	0.742	0.473	0.373	0.373	0.489	0.439	**0.161**
**Large**	**RMSE**	0.281	**0.273**	0.410	0.305	0.276	0.277	0.304	0.292	**0.220**
	**MAE**	0.373	**0.330**	0.695	0.426	0.340	0.341	0.439	0.391	**0.190**
Dixon-Price function										
**Small**	**RMSE**	0.456	**0.391**	0.439	0.418	0.392	0.391	0.417	0.414	**0.335**
	**MAE**	0.682	**0.519**	0.652	0.582	0.521	0.520	0.586	0.572	**0.225**
**Medium**	**RMSE**	0.431	**0.365**	0.425	0.396	0.365	0.365	0.393	0.388	**0.319**
	**MAE**	0.604	**0.440**	0.622	0.529	0.440	0.440	0.528	0.509	**0.224**
**Large**	**RMSE**	0.410	**0.348**	0.402	0.377	0.348	0.348	0.376	0.371	**0.303**
	**MAE**	0.551	**0.417**	0.569	0.494	0.417	0.417	0.497	0.476	**0.217**

According to Table 3, it can be observed that the errors of all surrogate models decrease as the training sample size increases. The performance of component surrogate models varies significantly depending on the specific problem. The SVR model exhibits better accuracy compared to the other two component surrogate models when approximating the Ackley function. However, when approximating the Extended Rosenbrock function and Dixon-Price function, the SVR model performs the worst, while the RBF model demonstrates higher accuracy compared to SVR and KRG. RBF model also shows the highest accuracy when approximating the Dixon-Price function, compared with the SVR and KRG models. The performance of the three individual surrogate models varies with the sample size and the function being approximated when approximating the Six-hump camel-back function and Hartman function.

Compared to all other surrogate models, the proposed DMEES method achieves higher accuracy in most cases. When approximating low-dimensional functions such as the six-hump camelback function and the Ackley function, the DMEES model exhibits smaller median values of MAE and RMSE at small sample sizes. Similarly, when approximating high-dimensional functions such as the Hartman function, Extended Rosenbrock function, and Dixon-Price function, the DMEES model consistently demonstrates small model errors regardless of the sample size. Particularly, when approximating the Extended Rosenbrock function and Dixon-Price function, regardless of the size of the training sample used, the median values for the error measurements of the DMEES model are significantly lower than those of other surrogate models. The median value of MAE for the DMEES model is close to half of the median value of MAE for other models, highlighting the significant accuracy advantage of the DMEES model.

**Fig 5:**
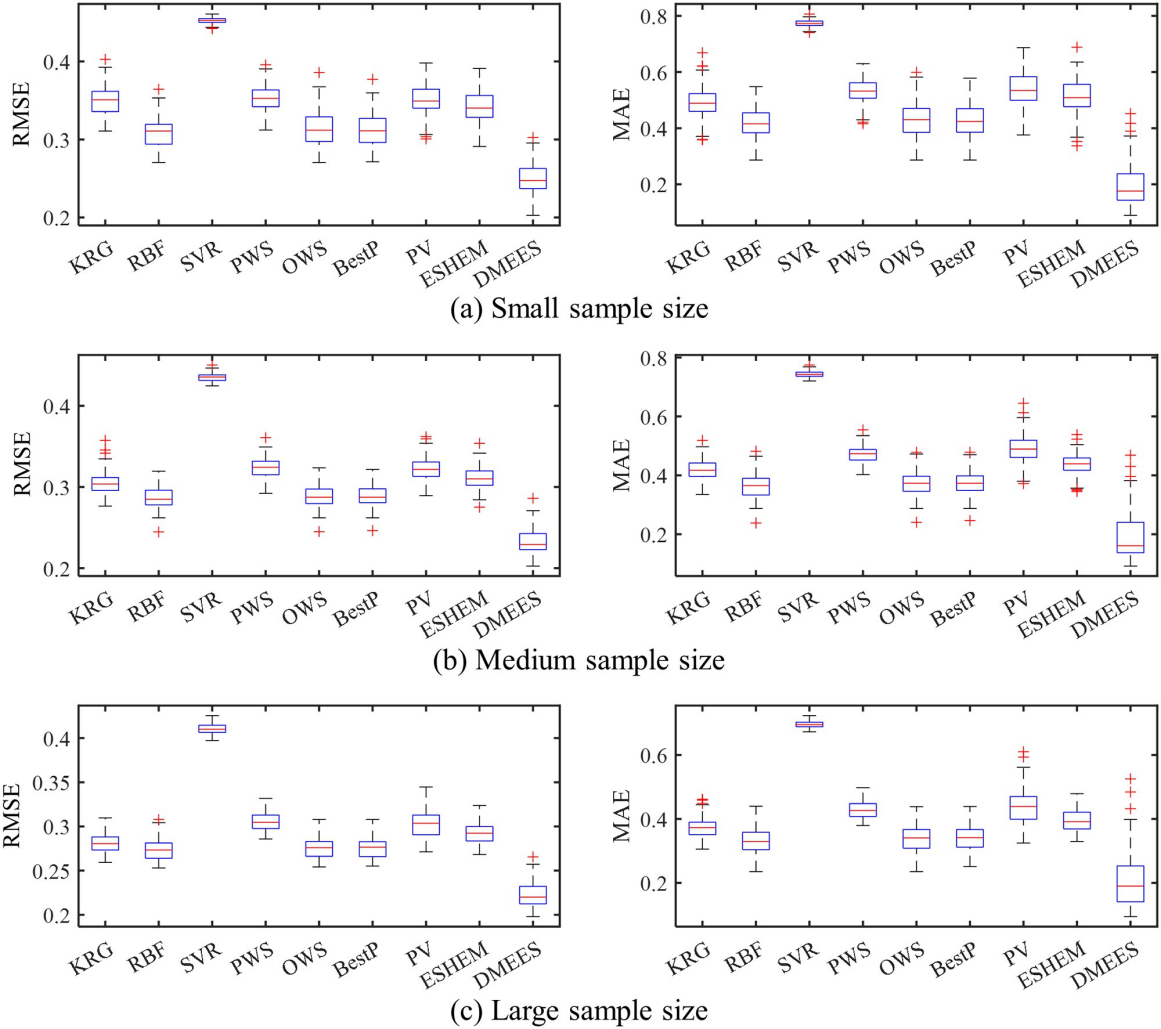
Boxplots of approximation errors for surrogate models when fitting the Extended Rosenbrock function. 100 tests of surrogate model are performed for each sample size. The training samples of each test are generated randomly. (a) RMSE and MAE distributions under small training samples. (b) RMSE and MAE distributions under medium training samples. (c) RMSE and MAE distributions under large training samples.

**Fig 6:**
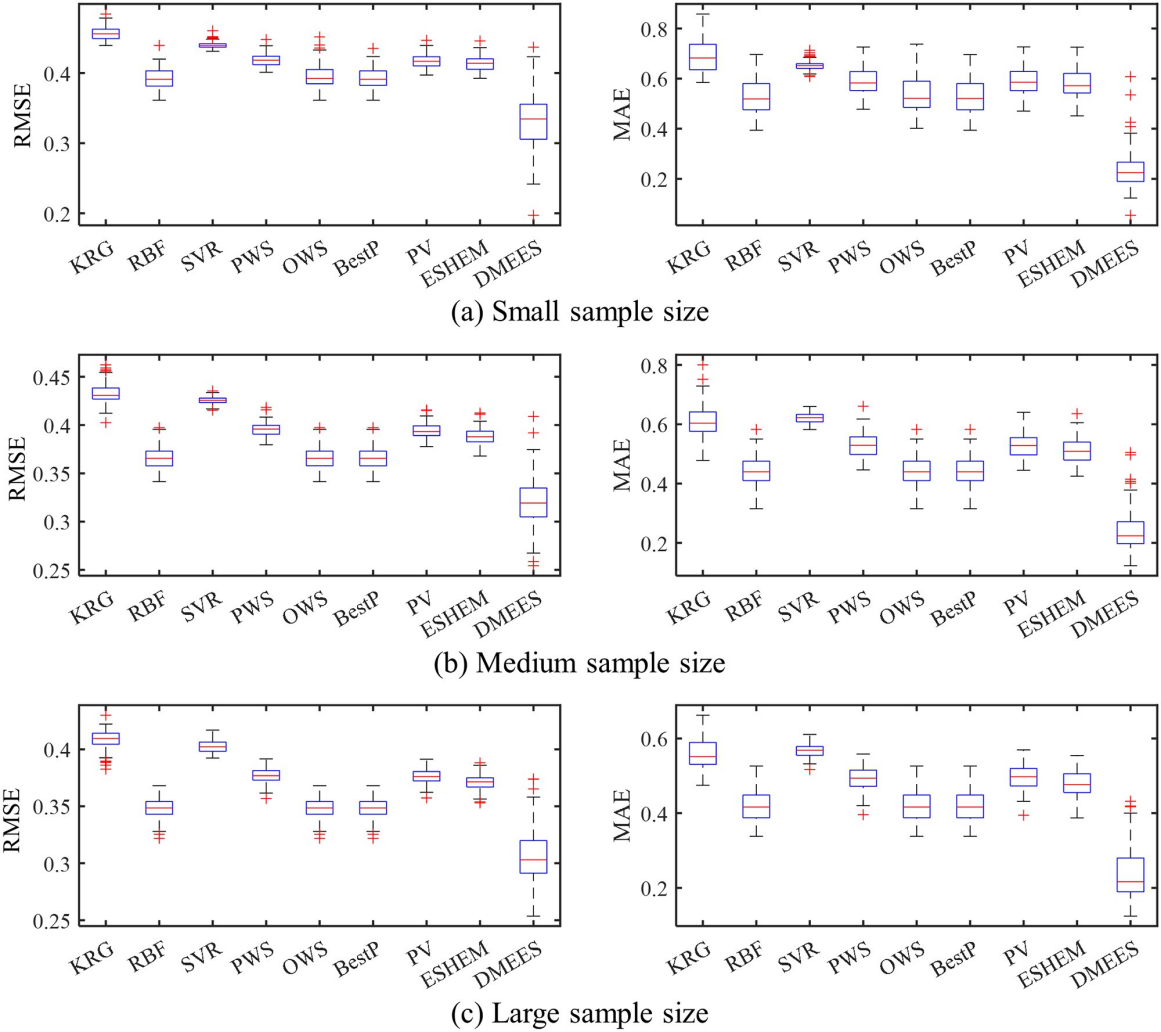
Boxplots of approximation errors for surrogate models when fitting the Dixon-Price function. 100 tests of surrogate model are performed for each sample size. The training samples of each test are generated randomly. (a) RMSE and MAE distributions under small training samples. (b) RMSE and MAE distributions under medium training samples. (c) RMSE and MAE distributions under large training samples.

The boxplots in Figs 5 and 6 respectively present the error distributions of surrogate models when approximating the Extended Rosenbrock function and Dixon-Price function. These boxplots are used to illustrate the error distribution characteristics of the surrogate models. The horizontal red line in the middle of the blue box in the graph represents the median value of approximation error of a surrogate model. The two horizontal blue lines of the box, from bottom to top, represent the 25^th^ percentile and 75^th^ percentile of the approximation error of a surrogate model, respectively. The lines extending above and below the box (whiskers) indicate the overall distribution of the approximation error of a surrogate model. Each red cross outside the range of the error distribution represents an outlier. The black horizontal lines at the ends of the whiskers represent the upper and lower values of the error, excluding outliers.

As shown in Fig 5, the MAE distribution of the DMEES model is more scattered compared to the other models. The wide distribution of MAE values suggests that, in terms of local accuracy, the DMESS model is more sensitive to training sample variations when fitting the Extended Rosenbrock functions compared to other models. The higher the sensitivity of a surrogate model to variations in the training sample, the worse its robustness. However, the distribution of RMSE values in Fig 5 indicates that the DMEES model has a similar level of robustness as the other surrogate models (except for SVR) from the perspective of global accuracy. As shown in Fig 6, the widespread distribution of MAE and RMSE indicates that, both from the perspective of global accuracy and local accuracy, the DMESS model is more sensitive to variations in the training sample than other models when fitting Dixon-Price function.

It also can be observed from the Figs 5 and 6 that when approximating the Extended Rosenbrock function and Dixon-Price function, regardless of the sample size, the 75^*th*^ percentile line of the DMEES model’s error is lower than the minimum error value (lower edge of the error box) of the other models. This implies that 100 model performance tests, the DMEES model achieves the minimum MAE and RSME in at least 75 of the tests. Therefore, it is evident that the DMEES model exhibits a significant accuracy advantage when approximating the Extended Rosenbrock function and the Dixon-Price function.

Based on the numerical test results, it is evident that the proposed DMESS model outperforms other surrogate models in terms of approximation accuracy. It’s important to note that, in some cases, the limitations in the robustness of DMESS should be considered. As mentioned in the introduction, the primary performance criterion for surrogate models is approximation accuracy. However, it is crucial to acknowledge that no surrogate model can achieve optimal performance for all problems under all evaluation criteria.

### Engineering case study

**Fig 7:**
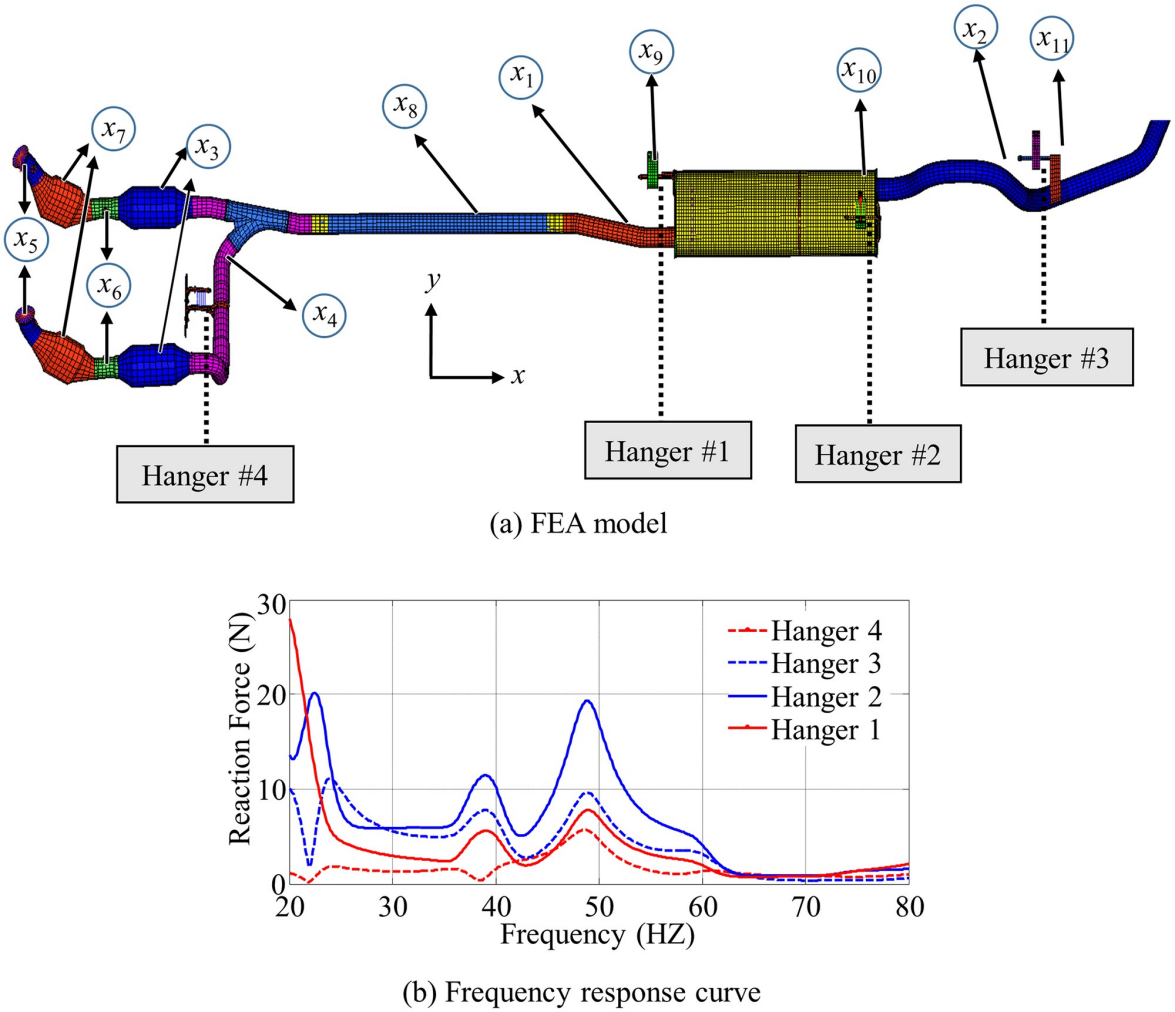
Frequency response analysis model and hanger force-frequency curve of automotive exhaust pipe system.

To validate the effectiveness of the DMEES model in solving engineering problems, an automotive exhaust pipe design case is used to test the approximation capability. Noise, vibration and harshness (NVH) performance is one of the most important factors directly affecting the driving experience of customers. A well-designed exhaust system plays a pivotal role in enhancing NVH performance [42]. In the optimization design of structural parameters for automotive exhaust systems, surrogate models are often used as approximations of computationally expensive finite element simulation models to improve efficiency [43–45]. In this case, the performance of the surrogate model is studied by approximating the mathematical relationship between the structural parameters of the exhaust system and the peak value of frequency response obtained from finite element analysis. Fig 7 shows the finite element model of the automotive exhaust pipe and the frequency response of the reaction forces at various hanger positions.

As shown in Fig 7 there are four hangers in the exhaust system, including the transmission bracket hanger, the muffler inlet hanger, the muffler outlet hanger, and the tailpipe hanger. Excitation from the engine causes the exhaust hangers to exert reaction forces on the chassis and body structure, which play an important role in evaluating the overall NVH performance of the vehicle. Since the frequencies of the vehicle at idle (35Hz to 40Hz), parked idle (40Hz to 50Hz), and non-idle (50Hz to 70Hz) are less than 80Hz, and the modal frequencies that cause vibration in the exhaust system will be higher than 20Hz, the Nastran software is used to calculate the reaction forces of the exhaust hangers on the chassis and body structure within the specific frequency range of 20Hz to 80Hz. An example of frequency responses for one design parameter setting is shown in Fig 7(b). The peak values of the reaction forces on the four hangers are labeled as *PF*_1_, *PF*_2_, *PF*_3_, and *PF*_4_, respectively.

The main design parameters of the exhaust pipe include the hanger locations and the thickness of the pipe. Fig 7(a) indicates 11 variables that include the hanger locations and the thickness of the pipe. Table 4 provides detailed information about these variables and their ranges. In this case, a surrogate model will be constructed to approximate the mathematical relationship between these design variables and the peak forces *PF*_1_, *PF*_2_, *PF*_3_, and *PF*_4_.

**Table 4:** Design variable information for an automotive exhaust pipes.

DV type	DV name	Description	Range (mm)
Thickness	*x*_1_	Tailpipe	[1.2,2.2]
	*x*_2_	Muffler Inlet	[1.2,2.2]
	*x*_3_	Upper Converter	[1.2,2.2]
	*x*_4_	Crossover Pipe	[1.2,2.2]
	*x*_5_	Catalyst Converter Inlet	[1.2,2.4]
	*x*_6_	Pipe between Cones	[1.2,2.4]
	*x*_7_	Lower Converter	[1.2,2.2]
	*x*_8_	Exit Pipe	[1.2,2.2]
Locations	*x*_9_	Position change of hanger 1	[-30,30]
	*x*_10_	Position change of hanger 2	[-30,30]
	*x*_11_	Position change of hanger 3	[-20,20]

Herein, 1000 sets of samples are generated by using OLHS based on the design variable ranges provided by Table 4. The simulation is then run to extract the peak forces corresponding to each sample, forming a sample data base with 1000 sets of samples. Randomly selected training samples are used to train the surrogate model, while the remaining samples are used as test samples to calculate the error metrics of the surrogate model. The number of training samples is set to 6*n*_*d*_ for the small sample case, 12*n*_*d*_ for the medium sample case, and 24*n*_*d*_ for the large sample case, where *n*_*d*_ is the number of independent variables. To avoid the contingency of test results, the test is repeated 100 times by using different random seeds setting.

**Fig 8:**
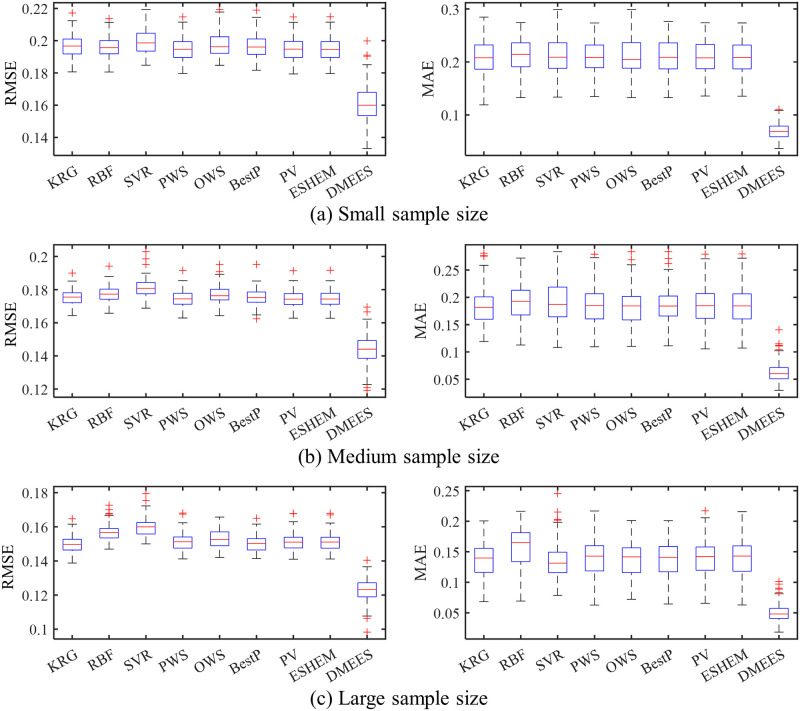
Boxplots of approximation errors for surrogate models when fitting *PF*_1_. 100 tests of surrogate model are performed for each sample size. The training samples of each test are generated randomly. (a) RMSE and MAE distributions under small training samples. (b) RMSE and MAE distributions under medium training samples. (c) RMSE and MAE distributions under large training samples.

**Fig 9:**
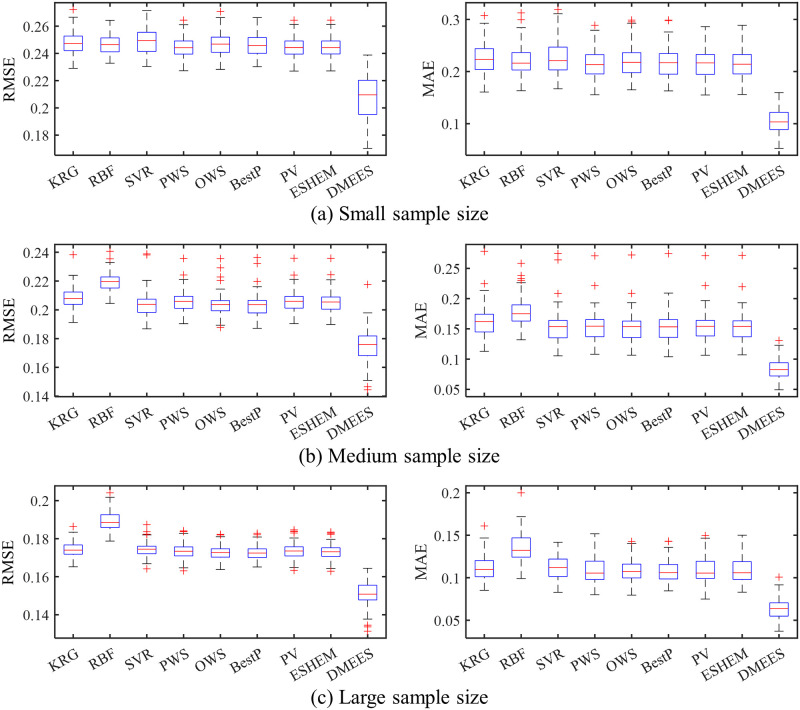
Boxplots of approximation errors for surrogate models when fitting *PF*_2_. 100 tests of surrogate model are performed for each sample size. The training samples of each test are generated randomly. (a) RMSE and MAE distributions under small training samples. (b) RMSE and MAE distributions under medium training samples. (c) RMSE and MAE distributions under large training samples.

**Fig 10:**
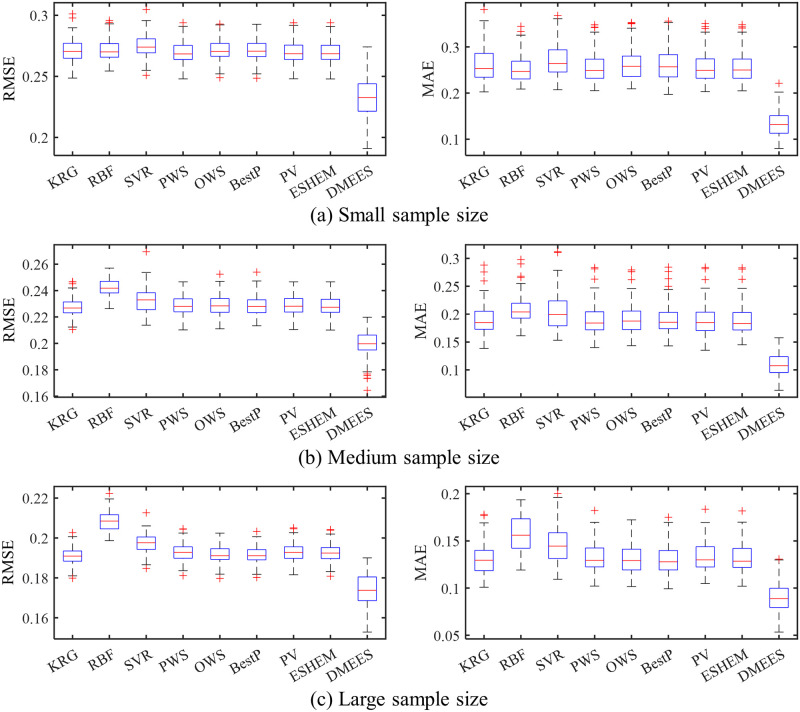
Boxplots of approximation errors for surrogate models when fitting *PF*_3_. 100 tests of surrogate model are performed for each sample size. The training samples of each test are generated randomly. (a) RMSE and MAE distributions under small training samples. (b) RMSE and MAE distributions under medium training samples. (c) RMSE and MAE distributions under large training samples.

**Fig 11:**
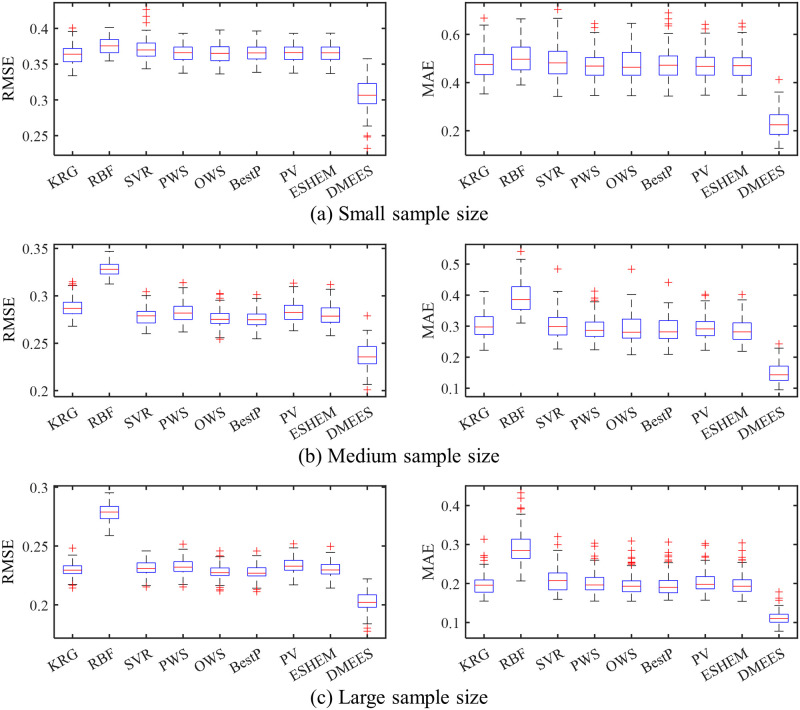
Boxplots of approximation errors for surrogate models when fitting *PF*_4_. 100 tests of surrogate model are performed for each sample size. The training samples of each test are generated randomly. (a) RMSE and MAE distributions under small training samples. (b) RMSE and MAE distributions under medium training samples. (c) RMSE and MAE distributions under large training samples.

Figs 8–11 provide the boxplots of the MAE and RMSE distributions of the surrogate models when approximating *PF*_1_, *PF*_2_, *PF*_3_, and *PF*_4_ under random testing. From these four figures, a decrease in errors for all surrogate models could be be observed as the training sample size increases. This is because with more training samples, the surrogate models can learn more, and their accuracy becomes higher. According to the distribution of the RMSE, the DMEES model exhibits relatively poorer robustness compared to other models from the perspective of global accuracy. However, when examining the distribution of the MAE values, considering the variations in the approximation targets and sample sizes, the robutness of the DMEES model is similar to that of other models in only a few cases, while in the majority of cases, the DMEES model demonstrates better robustness than the other models. Comparing accuracy and robustness, DMESS clearly outperforms other surrogate models.

From Figs 8–11, it can also be observed that when approximating *PF*_1_, *PF*_2_, *PF*_3_, and *PF*_4_, both in terms of RMSE and MAE, the 75^*th*^ percentile of DMEES model is lower than the minimum error of other models. This indicates that in at least 75 out of 100 tests, the DMEES model has the smallest error. In a few cases, such as the MAE boxplots in Fig 8(a) and 8(b), and the RMSE boxplot in Fig 9(c), the upper edge of the DMEES model’s boxplot is lower than the lower edge of the boxplot corresponding to other surrogate models. This suggests that in each testing instance, the DMEES model consistently achieves the minimum error value. Therefore, it can be concluded that the DMEES model has a significant accuracy advantage.

## Conclusion

This paper presents a novel adaptive-weight ensemble surrogate model based on the principles of distance and mixture error. The new ensemble surrogate model takes into account the spatial relationship between prediction samples and training samples, along with the intricate considerations of mixture error within constituent surrogate models. Specifically, the initial weights of the component surrogate models are determined by both global and local errors, in conjunction with the distance relationship between the sample to be predicted and the training samples. Additionally, a weight adjustment factor is employed to fine-tune the initial weight of the component surrogate model. This adjustment enhances the prediction accuracy of the proposed adaptive-weight ensemble surrogate model, especially in proximity to the training samples. Advantages of the proposed method were demonstrated by five highly nonlinear benchmark functions and an engineering finite element model. The analysis results from numerical and engineering cases lead to the following conclusions.

1. Both the numerical and engineering case studies demonstrate that the proposed DMEES model outperforms other surrogate models in terms of predictive accuracy.
2. The proposed DMEES model demonstrates strong robustness in specific scenarios but shows reduced robustness compared to alternative surrogate models in other cases, as measured by local accuracy. This conclusion also applies to model robustness when measured by global accuracy.
3. While no surrogate model can be universally applicable to all problems across every evaluation criteria, the proposed DMEES model consistently exhibits superior predictive accuracy across a wide range of performance evaluation metrics.

These results underscore the significant advantages of our approach and its promising potential for optimizing surrogate-based structural design.

## Supporting information

S1 Data (ZIP)
