# Supplementary material for: A novel adaptive-weight ensemble surrogate model base on distance and mixture error
Source: PLoS One. 2023 Oct 31;18(10):e0293318. doi: 10.1371/journal.pone.0293318 (PMC10617703; doi:10.1371/journal.pone.0293318)
Supplement: S1 Data — (ZIP) [file pone.0293318.s001.zip › meta data for fig4 and 7/Figure 4.pptx]

## Slide 1
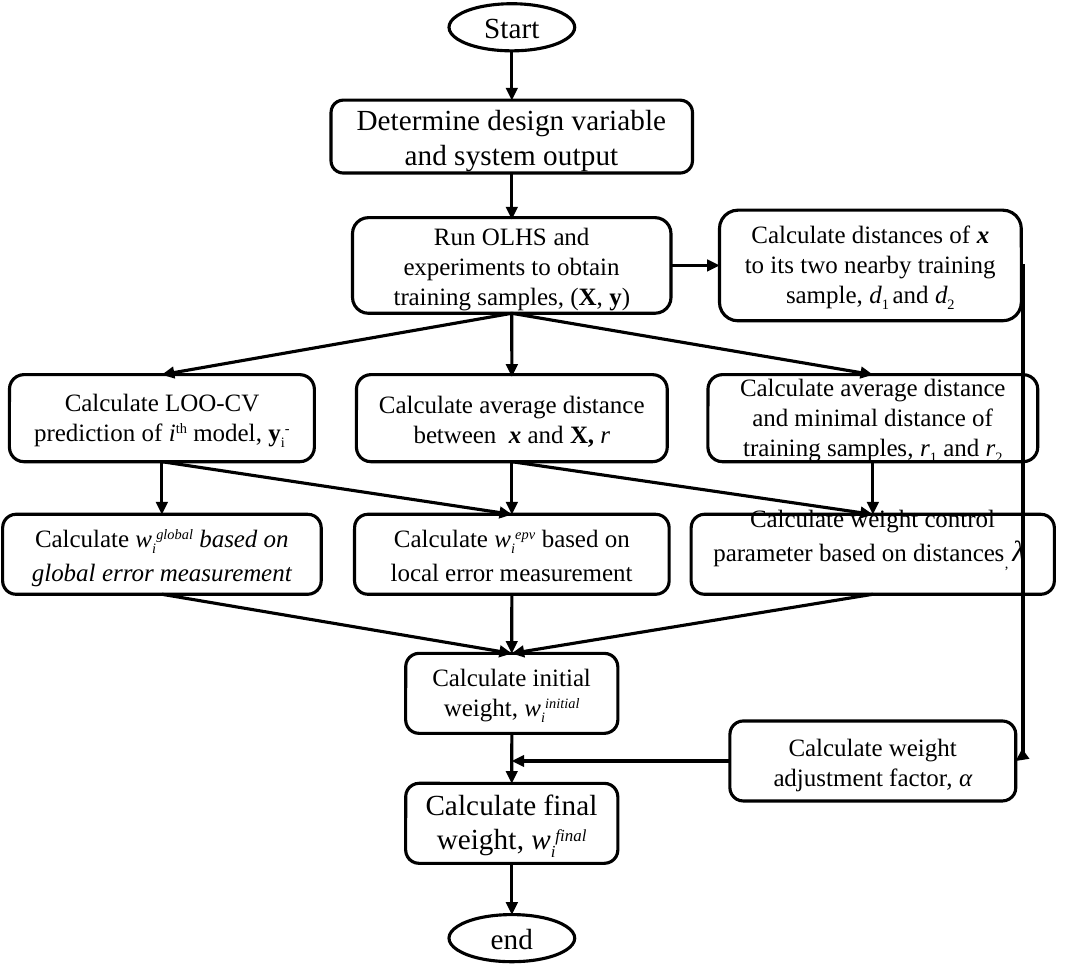

Start
Determine design variable and system output
Calculate distances of x to its two nearby training sample, d1 and d2
Run OLHS and experiments to obtain training samples, (X, y)
Calculate LOO-CV prediction of ith model, yi-
Calculate average distance between x and X, r
Calculate average distance and minimal distance of training samples, r1 and r2
Calculate wiglobal based on global error measurement
Calculate wiepv based on local error measurement
Calculate weight control parameter based on distances, λ
Calculate initial weight, wiinitial
Calculate weight adjustment factor, α
Calculate final weight, wifinal
end
