# Supplementary material for: A novel adaptive-weight ensemble surrogate model base on distance and mixture error
Source: PLoS One. 2023 Oct 31;18(10):e0293318. doi: 10.1371/journal.pone.0293318 (PMC10617703; doi:10.1371/journal.pone.0293318)
Supplement: S1 Data — (ZIP) [file pone.0293318.s001.zip › meta data for fig4 and 7/Figure 7.pptx]

## Slide 1
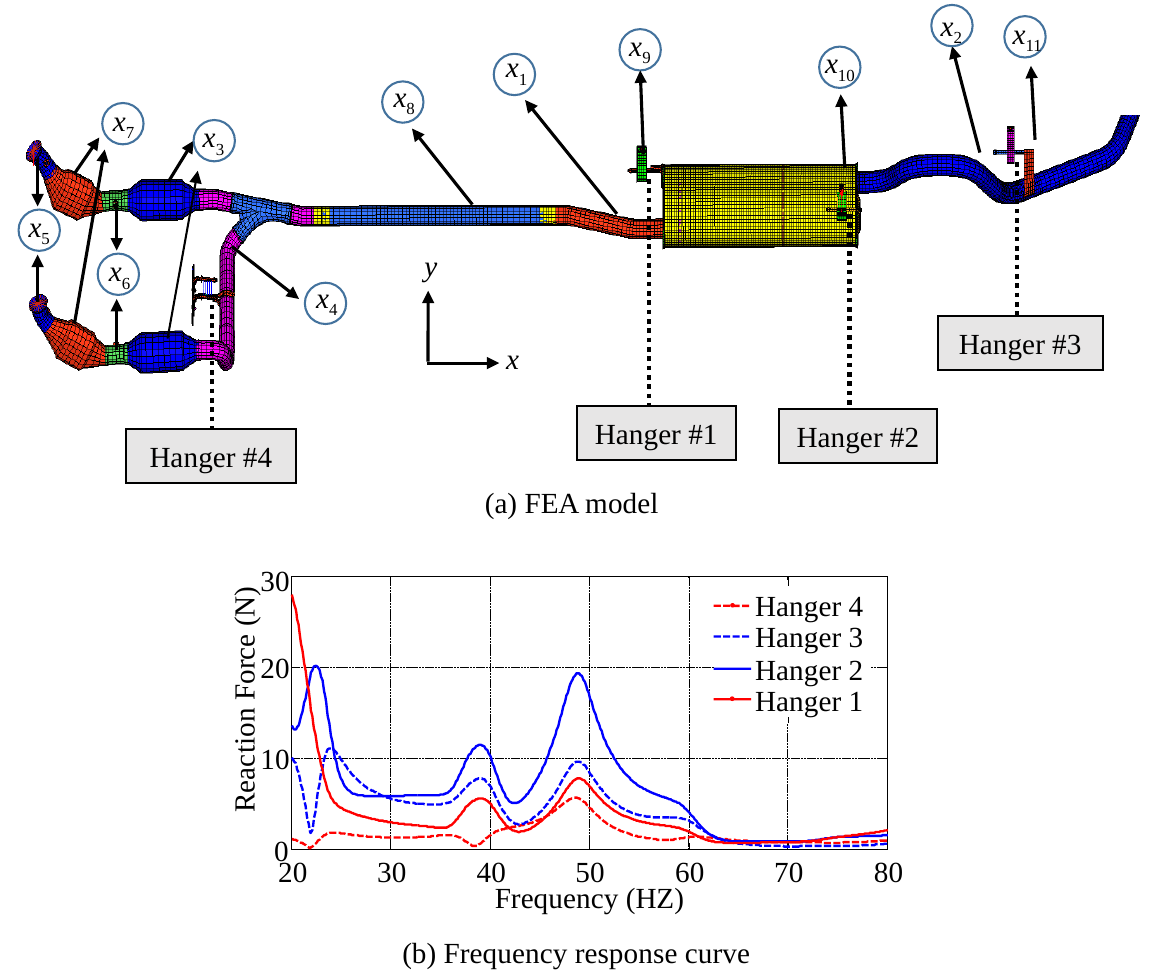

x2
x11
x9
x10
x1
x8
x7
x3
x5
y
x6
x4
Hanger #3
x
Hanger #1
Hanger #2
Hanger #4
30
Hanger 4
Hanger 3
20
Hanger 2
Reaction Force (N)
Hanger 1
10
0
20
30
40
50
60
70
80
Frequency (HZ)
(a) FEA model
(b) Frequency response curve
